# Prevalence of Mild to Moderate Mental Illness, Correlates of Treatment Patterns, and Perceived Unmet Need among U.S. Adults: Results from the National Survey on Drug Use and Health, 2021

**DOI:** 10.2174/0117450179394114251013063017

**Published:** 2025-11-21

**Authors:** Valerie L. Forman-Hoffman, Megan Flom, Andre Williams, Timothy Mariano

**Affiliations:** 1 Woebot Health, San Francisco, CA, USA; 2 RR&D Center for Neurorestoration and Neurotechnology, VA Providence Healthcare System, Providence, RI, USA; 3 Department of Psychiatry and Human Behavior, Warren Alpert Medical School of Brown University, Providence, RI, USA

**Keywords:** Mental health prevalence, Mild to moderate mental illness, Correlates, Epidemiology, Barriers to care, Pandemic

## Abstract

**Purpose:**

Mental health issues continue to affect millions despite the availability of evidence-based treatment. The burden of illness and associated characteristics of mild to moderate mental illness (MMMI) among community-dwelling U.S. adults has not been reported in the peer-reviewed literature to date.

**Methods:**

Analyses of the cross-sectional 2021 National Survey on Drug Use and Health (NSDUH) conducted across the U.S. allowed for the estimation of the prevalence and correlates of MMMI as well as of the overall and virtual treatment landscape, perceived unmet need, and barriers to care.

**Results:**

Nearly 44 million community-dwelling U.S. adults (17.2%), about 1 in 6, are estimated to have past-year MMMI, of whom 41.3% report mental health treatment receipt. Over 62% of those who received mental health treatment did so virtually.

**Discussion:**

MMMI commonly affected U.S. adults; a majority are not getting treatment. Several correlates of MMMI and treatment receipt might point to demographic and clinical groups in need to additional services.

**Conclusion:**

This study is the first of its kind to report nationally representative estimates and correlates of MMMI among community-dwelling U.S. adults. Despite the widespread use of virtual services when accessing mental health treatment, findings suggest the need for access to low-cost, easily accessible, on-demand mental health services to better serve adults with MMMI.

## 1 INTRODUCTION

Mental health issues affect millions of adults nationwide [1]. Nearly half of adults will meet criteria for one or more diagnosable mental disorders over the course of a lifetime [2]. Despite the availability of evidence-based treatments, less than half of those with any mental illness (AMI) report receiving treatment in the past year [1]. Treatment gaps exist due to many barriers to accessibility and delivery of care. Coupled with a nationwide shortage of mental health providers to meet the need of those with mental health issues [3, 4], adults cite cost, limited time to seek and receive care, not knowing where to go to receive services, and not having transportation to travel when they are able to make an appointment, stigma, and not believing treatment is necessary as barriers to care receipt [1]. In addition to the increased prevalence and demand for mental health services during the COVID-19 pandemic [5], other barriers to access were created by the fear of transmission [6], as healthcare, including mental health services, shifted toward increased virtual delivery [7].

Since the advent of the pandemic, the prevalence as well as the appropriateness and effectiveness of the virtual delivery of mental health services, including for those with mental health concerns accompanied by more severe forms of impairment, have been demonstrated [8-10]. It is undeniably important to study serious mental illness (SMI) as a separate category of individuals with unique characteristics and associated treatment needs, given levels of impairment and disparities experienced [11]. Indeed, the Substance Abuse and Mental Health Services Administration (SAMHSA) sponsors a nationally representative, annual survey, the National Survey of Drug Use and Health (NSDUH), to estimate the past-year prevalence of United States (U.S.) adults living with SMI, in part to assist with planning for services at the state level [12]. The algorithms created for use with NSDUH data also allow for the estimation of AMI. Although SAMHSA annually releases detailed tables reporting the past-year prevalence of individuals with AMI but not SMI (*i.e.*, mild to moderate mental illness; MMMI), as well as the prevalence of MMMI and its treatment landscape (including the perceived unmet need for mental health services), these reports have not, to the authors’ knowledge, been distributed in the peer-reviewed literature, setting the stage for the current study. Since this study is exploratory, no specific hypotheses were tested.

Although studies have reported a net benefit of treatment for those with MMMI [13], a finer-grained understanding of this population, its characteristics, and its needs will help optimize treatment equity and capacity. This is especially true given the recent proliferation of virtual services, such as telehealth and digital mental health interventions (DMHIs), since the pandemic, which could potentially benefit those with MMMI and allow in-person services to be reserved for those with higher acuity needs. In addition, the prevalence correlates specific to MMMI have not been reported, precluding clarity about groups of adults suffering disparities. Identifying adults with increased risk of MMMI and groups less likely to get treatment and more likely to perceive an unmet need for care is an important first step towards improving mental health equity.

## 2 METHODS

### 2.1 Survey Design, Setting, and Participants

NSDUH is an annual, cross-sectional survey sponsored by SAMHSA of adolescents and adults aged 12 and older in the U.S., representative at the national and state levels of those living in the community, including households and noninstitutional group settings (*i.e.*, excluding individuals who are homeless and not living in shelters, incarcerated, residing in nursing homes, institutionalized, hospitalized in long-term care facilities, or serving on active military duty). At the time of study conception, the most recently released NSDUH data available *via* a public-use file [14] was from 2021. As this is a publicly available dataset, ethical approval and consent for this secondary analysis were not required. The Sex and Gender Equity in Research (SAGER) Guidelines were followed by the authors.

### 2.2 Mental Illness Assessment

Adults aged 18 or older were assessed by trained interviewers, either in person or online, and classified as having any mental illness (AMI) if they had any mental, behavioral, or emotional disorder in the past year, in accordance with criteria from the Diagnostic and Statistical Manual of Mental Disorders, 4th edition (DSM-IV), excluding developmental disorders and substance use disorders [15]. Data from a subset of NSDUH adult respondents interviewed between 2008 and 2012 were used to develop statistical models that enabled classification of past-year mental illness status, along with the level of impairment severity, among adults interviewed in the 2021 NSDUH [16]. Those designated as having past-year AMI were further classified as having serious mental illness (SMI) if there was substantial interference with one or more major life activities.

Those classified with mild to moderate levels of impairment (MMMI) consisted of adults with AMI who did not meet SMI criteria. The AMI model’s receiver operating characteristic (ROC) analyses yielded a model that included five predictors, with a sensitivity of 0.569, specificity of 0.906, and an area under the curve (AUC) of 0.738; the SMI model had a sensitivity of 0.509, specificity of 0.980, and an AUC of 0.745 [17]. Additional methodological details on NSDUH classification of mental illness are described elsewhere [18]. Data from the 2021 NSDUH public use files, including 9,594 adults aged 18 or older classified as having past-year MMMI, were used for this study’s analyses. The NSDUH public use file is a de-identified, publicly available dataset.

### 2.3 Additional Variables Studied

Past-year mental health treatment was assessed *via* questions about 1) inpatient care including services received at a psychiatric hospital, a general hospital psychiatric unit or medical unit for mental health treatment, or another type of hospital for mental health care, 2) outpatient care received at an outpatient mental health center or clinic, the office of a private therapist (psychologist, psychiatrist, social worker, or counselor), a non-clinic doctor’s office, an outpatient medical clinic for mental health care, a partial day or day treatment program for mental health care, a school or university setting clinic or center, or another type of facility for mental health care, 3) the use of a prescription medication for a mental health problem, and 4) a virtual mental health care visit (*i.e.*, over the phone, by email, or through video calling). The NSDUH surveys also included questions regarding perception of having an unmet mental health care need in the past year. Those who perceived an unmet need and did not report receiving treatment were also asked to report reasons, or barriers, for why they did not get any mental health care. Model covariates were selected from all available variables and included those theoretically related to one of the outcomes examined. These included age, sex, race/ethnicity, employment status, county type (as designated by metropolitan statistical area population size), poverty level (based on annual household income), education, health insurance status, perceived overall health status, sexual identity, and veteran status, as recommended by published guidelines [19].

### 2.4 Statistical Analysis

Item response rates in the NSDUH were high; however, the public use file included imputed values for missing data [20]. Weight, stratum, and cluster variables in the dataset were used to estimate the weighted numbers and percentages of each variable studied. Bivariate associations between covariates and mental health variables were assessed *via* chi-square statistics, with *P*<0.05 considered statistically significant. Logistic regression models including all covariates were used to study adjusted associations, reported as adjusted odds ratios (ORs) and 95% confidence intervals (CIs), noting statistical significance. All analyses were conducted using R statistical software (version 4.3.1) to account for the complex sample design and sampling weights of the survey data [21].

## 3 RESULTS

### 3.1 Prevalence and Correlates of Mild to Moderate Mental Illness

An estimated 43.6 million U.S. adults (17.2% of all adults and about three-quarters of those with AMI) were classified as having past-year mild to moderate mental illness (MMMI) in 2021 (Table **1**). All assessed covariates except county type were significantly correlated with MMMI in bivariate analyses. In adjusted models, MMMI was associated with all covariates except county type and veteran status. Specifically, adults with higher prevalence of MMMI included those aged 18-25 years (versus 50-64 years or 65 years and older), females (versus males), adults identifying as non-Hispanic or Latino more than one race (versus non-Hispanic or Latino white), adults living at less than 100% of the poverty level (versus 200% or more), adults covered by Medicaid or Chip insurance (versus private insurance), and college graduates (versus adults with lower education levels). Adults with lower prevalence of MMMI included adults identifying as non-Hispanic or Latino black or non-Hispanic Asian or Hispanic or Latino (versus non-Hispanic white or Latino white), full-time employees (versus part-time employed or unemployed adults), adults rating their overall health as excellent (versus lower ratings), and adults identifying as heterosexuals (versus adults identifying as lesbian or gay, bisexual, or not knowing/refusing to answer sexual orientation). After re-running the bivariate chi-square analyses and adjusted regression model removing adults with SMI from the comparison group to create a more homogeneous “no mental illness” comparator, the patterns of significant correlates with MMMI remained the same (data not shown), with two exceptions: (1) adults aged 26–49 were also less likely than those aged 18–25 years to have past-year MMMI, and (2) adults identifying as non-Hispanic or Latino Native Hawaiian or Pacific Islander were less likely than non-Hispanic or Latino white adults to have past-year MMMI.

### 3.2 Mental Health Treatment Prevalence and Correlates Among those with MMMI

Approximately 17.5 million U.S. adults with MMMI reported past-year receipt of mental health treatment (41.3%) (Table **2**). Age, sex, race/ethnicity, employment, education, health insurance, and sexual identity were significantly correlated with mental health treatment in bivariate analyses. Adjusted analyses revealed that females (versus males), those employed part-time or in “other” employment (versus full-time), individuals with good/fair/poor health status (versus excellent), and bisexual individuals (versus heterosexual) were more likely to report receiving past-year mental health treatment. Conversely, non-Hispanic Black, non-Hispanic Asian, and Hispanic adults (versus non-Hispanic white), those with a high school education or less (versus college graduates), and those with no insurance (versus other insurance types) were less likely to report receiving past-year mental health treatment.

Nearly 2 percent of adults with past year MMMI reported receiving inpatient services, 18.6% received outpatient services, 30.5% received a prescription mental health medication, and 25.8% received mental health treatment virtually (Table **3**). Of those who received past-year mental health treatment, a majority did so virtually (62.6%). Nearly three-quarters (74.1%) reported a mental health-related medication prescription. Less than half (45.8%) received outpatient mental health treatment, and almost 5 percent (4.6%) received inpatient treatment.

Significant correlates of receiving virtual mental health services among adults with MMMI included age, sex, race/ethnicity, county type, education, health insurance, and sexual identity in bivariate analyses. In adjusted analyses, females (versus males), part-time and “other” employed (versus full-time), bisexual (versus heterosexual), and veterans (versus those never having served in the armed forces) were more likely to report receiving virtual mental health services in the past year. Also, non-Hispanic black, non-Hispanic Asian, and Hispanic (versus non-Hispanic white), those living in a small or no metropolitan area county (versus those living in large), with a high school or less education (versus college graduates), and those with no insurance (versus other insurance types) were less likely to report receiving virtual mental health services in the past year (data not shown).

### 3.3 Perceived Unmet need and Barriers to Care Among those with MMMI

An estimated 8.4 million adults with MMMI (19.9%) perceived an unmet need for mental health treatment in the past year. More specifically, 23.6% of those who received past-year mental health care perceived an unmet need compared to 17.3% among those who did not receive past-year care. Correlates of having any perceived unmet need among all adults with MMMI (*i.e.*, regardless of past-year treatment status) included age, sex, race/ethnicity, employment, county type, education, health insurance, overall health, and sexual identity in bivariate analyses. In adjusted analyses, females (versus males), non-Hispanic adults reporting more than one race (versus non-Hispanic white adults), those with good (versus excellent) self-reported health, and bisexual (versus heterosexual) adults with MMMI and no past-year mental health treatment reported being more likely to perceive an unmet need. Adults aged 26 and older (versus those aged 18–25), those identifying as non-Hispanic Native Hawaiian or Pacific Islander or Hispanic (versus non-Hispanic white), individuals employed in “other” types of work (versus full-time employment), those living in non-metropolitan areas (versus large metropolitan areas), and those with a high school education or less (versus college graduates) were less likely to report perceived unmet need (data not shown).

Nearly all adults with MMMI (99%) with a perceived unmet need reported at least one barrier to care in the past year. The most prevalent barriers to mental health treatment included not being able to afford it (40.1%), not knowing where to go for services (31.1%), thinking they could handle the issues without treatment (27.1%), and not having time to get treatment (20.4%) (Table **4**).

## 4 DISCUSSION

Results from this study indicate that nearly 44 million community-dwelling U.S. adults were classified as having an MMMI in 2021, representing about 1 in 6 adults living in the household population captured by NSDUH survey methods. This is in addition to the 14 million classified as having SMI [22]. To the authors’ knowledge, this is the first report in the peer-reviewed literature of the national prevalence of MMMI. Despite the proliferation of new modalities for delivering mental health treatment, such as synchronous or asynchronous telehealth, only about 2 in 5 adults with MMMI reported receiving past-year mental health treatment in 2021. Notably, among those with MMMI who did receive care, over 3 in 5 received it virtually.

The year 2021 was at the height of the COVID-19 pandemic, which may have influenced the choice of treatment setting. Nonetheless, 2022 NSDUH data released after this study’s analyses indicated that a comparable proportion of those with MMMI who received treatment also did so virtually [1]. The trend toward increased use of virtual services for mental health treatment since the pandemic is likely to endure, and the future treatment landscape will probably include a mix of telehealth modalities (*e.g.*, treatment or counseling via phone, email, or videoconference) and mobile technologies, such as DMHIs. However, receipt of treatment does not necessarily indicate the adequacy of the services received. Therefore, additional studies are needed to investigate the nature of the treatment, particularly the types of virtual services being utilized.

Although some questioned the helpfulness of treating those with milder levels of mental illness severity, heightened risk for later negative clinical outcomes among even milder cases has been recognized [13]. Importantly, about 1 in 5 of those with MMMI, representing over 8 million adults, indicated a perceived unmet need for such services. This proportion was higher among those who had received past-year care than among those who had not (23.6% *vs.* 17.3%). These findings are in line with prior work demonstrating that perceived unmet need likely drives care-seeking [23] and that those receiving treatment may still perceive that their overall needs are not being met by current standards of care [24]. The most prevalent barriers noted among those with MMMI who perceived an unmet need, mainly cost, time, and inability to know where to go for care, suggest a possible role for newer care modalities such as easily accessible, low-cost DMHIs that can be used on demand at any time.

The sociodemographic correlates of mental health-related factors identified by adjusted analyses in this study highlight important disparities that still exist. Younger adults aged 18–25 years, females, those identifying as non-Hispanic or Latino with more than one race, part-time or unemployed individuals, those living below 100% of the poverty line, those with Medicaid insurance, those in less than excellent health, and individuals not identifying as heterosexual had higher prevalence estimates of MMMI than their respective reference categories. Interestingly, college graduates had higher past-year prevalence estimates compared with adults with lower levels of education. Although recent evidence points to attendees of higher education institutions having higher rates of mental disorders than their counterparts not attending school, some research has determined that these differences did not persist into later adulthood [25, 26]. Other studies have reported contradictory findings; however, factors such as having more stressful jobs are a potential reason for higher distress among more educated subgroups (see World Health Organization, n.d. for more information [27]).

Among those with MMMI, males had a lower prevalence of past-year mental health service receipt compared to females, which is important given the apparent rising prevalence in this population [1]. However, females with MMMI were more likely than males to report a perceived unmet need for treatment. One possible reason for this disparity could be stigma, which some have explored as contributing to differing treatment receipt rates between males and females [28, 29]. Other sociodemographic groups, such as adults identifying as Hispanic, displayed a similar pattern: they were less likely to receive care but also less likely to perceive an unmet need for it, potentially for similar reasons [30]. Conversely, adults with MMMI identifying as bisexual were more likely to receive past-year care but also more likely to perceive an unmet need, compared to adults identifying as heterosexual. Recent studies on the distinctive needs of sexual minorities suggest unique experiences of mental health issues, treatment receipt, and perceived unmet care [31].

Other important population groups with MMMI, such as non-Hispanic Black, non-Hispanic Asian individuals, and those with no insurance, had lower prevalence of past-year treatment, while groups like younger adults aged 18–25 years had higher unmet need among those not receiving past-year care. These findings replicate prior epidemiological research demonstrating care disparities that adversely affect traditionally underserved groups [24, 32].

Barriers to mental health care reported by at least 1 in 5 adults with MMMI who perceived an unmet need for mental health services include cost, time, not knowing where to go, and believing they could handle issues on their own. The deepening mental health crisis in the U.S [33]. underscores the urgent need for new ways to access and receive mental health care by those in need. Innovations in policy, public health, and technology will likely all be necessary to address this crisis. Regarding the latter, carefully curated DMHIs have been developed with demonstrated benefits that appear to outweigh tested harms [34, 35]. Continued innovation of these solutions may help reduce the nation’s mental health burden.

## 5 LIMITATIONS

The findings from this study should be considered in light of several important limitations resulting from the use of secondary data collected as part of a cross-sectional national surveillance effort, which does not permit exploration of causal relationships [36]. First, the sample included only adults living in households or noninstitutionalized group settings, excluding populations with some of the highest mental health needs, such as homeless individuals, long-term hospitalized patients, active-duty military personnel, and incarcerated adults [37]. Thus, these findings do not represent the complete picture of the U.S. mental health landscape, but only that experienced by community-dwelling adults. Second, mental illness categorization relied on a statistical model developed using 2008–2012 data rather than direct clinical interviews. Nonetheless, these models were carefully calibrated against interviewer-assessed DSM-IV criteria-based mental disorders [38] and continue to serve as the method used by states to quantify mental health block grant allocations issued annually in the U.S [12].

In the absence of nationally representative psychiatric epidemiological data to enable quantification of MMMI since the National Comorbidity Survey-Replication was conducted several decades ago [13], these data are widely used to quantify the mental health needs of the U.S. adolescents and adults. It, therefore, is unknown how the update to DSM-5 could affect these estimates [39]. Furthermore, SAMHSA refers to virtual mental health services in the NSDUH as essentially synonymous with telehealth to “include treatment/counseling for mental health, emotions, or behavior over the phone, by email, or through video calling” [21]. This definition makes it unclear how survey respondents should consider technologies such as DMHIs which may not require a direct line of communication between user and healthcare professional. Finally, this study relied on self-reported data, which potentially introduced social desirability and recall biases. In 2021, however, the NSDUH offered multimodal participation via web and, if preferred, in-person interviews [12].

In the absence of nationally representative psychiatric epidemiological data to enable quantification of MMMI since the National Comorbidity Survey-Replication was conducted several decades ago [13], these data are widely used to quantify the mental health needs of U.S. adolescents and adults. It, therefore, is unknown how the update to DSM-5 could affect these estimates [39]. Furthermore, SAMHSA refers to virtual mental health services in the NSDUH as essentially synonymous with telehealth, including “treatment/counseling for mental health, emotions, or behavior over the phone, by email, or through video calling” [21]. This definition makes it unclear how survey respondents should consider technologies, such as DMHIs, which may not require a direct line of communication between the user and healthcare professional. Finally, this study relied on self-reported data, which potentially introduced social desirability and recall biases. In 2021, however, the NSDUH offered multimodal participation *via* web and, if preferred, in-person interviews [12].

## CONCLUSION

This study is likely the first of its kind to report nationally representative estimates and correlates of MMMI in the U.S. community-dwelling adult population. In 2021, MMMI affected over 46.7 million American adults, with only 41.3% receiving mental health treatment over the course of the year. In addition to the majority using prescription medications, the receipt of virtual services was reported by over 3 in 5 of those who received past-year treatment, which was greater than the proportion using non-virtual outpatient services. A total of 8.4 million adults reported an unmet need for services, with the most frequently cited barriers being cost, time, not knowing where to go for services, and thinking they could handle the issues on their own. Significant associations with MMMI, the receipt of treatment, and/or having a perceived unmet need for care were found across varying sexes, race/ethnicities, education levels, sexual orientation categories, and insurance status, suggesting that continued efforts to decrease these inequities are needed. Findings suggest the need for additional low-cost, easily accessible, on-demand mental health services to better meet the needs of adults with MMMI.

## Figures and Tables

**Table 1:** Past-Year Mild to Moderate Mental Illness by Individual Characteristics among Adults Aged 18+.

	**Past Year Mild to Moderate Mental Illness^1^** **(Weighted Percent)**	**Bivariate Chi-Square** **(df)^2^, p value**	**Adjusted Logistic Regression Model Odds Ratio^3^** **(95% CI)**
**TOTAL**	Population N=43,682,795 Sample n=9,594 Weighted %=17.2%	N/A	N/A
**Age Category**			
18-25	22.2%	Chisq (3) = 701.41, p<.0001	REF
26-49	21.0%		0.93 (0.85, 1.02)
50-64	14.5%		**0.57 (0.49, 0.67)**
65 or Older	10.3%		**0.37 (0.30, 0.46)**
**Sex**			
Male (1)	14.2%	Chisq (1) = 279.37, p<.0001	REF
Female (2)	20.0%		**1.45 (1.29, 1.63)**
**Race/Ethnicity**			
Not Hispanic or Latino White (1)	17.9%	Chisq (6) = 131.66, p<.0001	REF
Not Hispanic or Latino Black/African American (2)	17.2%		**0.79 (0.67, 0.93)**
Not Hispanic or Latino Native American or Alaskan Native (3)	14.8%		0.58 (0.31, 1.08)
Not Hispanic or Latino Native Hawaiian or Pacific Islander (4)	11.6%		0.52 (0.26, 1.02)
Not Hispanic or Latino Asian (5)	13.1%		**0.60 (0.48, 0.76)**
Not Hispanic or Latino More than One Race (6)	27.5%		**1.36 (1.02, 1.85)**
Hispanic or Latino (7)	15.3%		**0.68 (0.58, 0.79)**
**Employment**			
Full-time (1)	16.8%	Chisq (3) = 198.23, p<.0001	REF
Part–time (2)	21.8%		**1.31 (1.12, 1.52)**
Unemployed (3)	23.6%		**1.32 (1.10, 1.59)**
Other (4)	15.4%		0.98 (0.87, 1.11)
**County Type**			
Large Metro (1)	17.2%	Chisq (2) = 6.604, p=0.4327	REF
Small Metro (2)	17.7%		0.98 (0.86, 1.13)
Non-Metro (3)	16.3%		0.88 (0.73, 1.05)
**Poverty Level**			
<100% poverty threshold (1)	21.6%	Chisq (2) = 154.92, p<.0001	**1.21 (1.04, 1.40)**
100-199% poverty threshold (2)	18.7%		1.14 (0.97, 1.35)
200+% poverty threshold (3)	15.7%		REF
**Education**			
< High School (1)	15.8%	Chisq (3) = 83.997, p<.001	**0.67 (0.54, 0.83)**
High School Graduate (2)	15.0%		**0.65 (0.56, 0.75)**
Some College (3)	18.8%		**0.84 (0.75, 0.94)**
College Graduate (4)	18.1%		REF
**Health Insurance^4^**			
Medicare	12.7%	Chisq (5) = 257.05, p<.0001	1.19 (0.88, 1.60)
Medicaid or CHIP	22.4%		**1.23 (1.05, 1.44)**
Tricare, Champus, ChampVA, VA, or Military	19.8%		1.17 (0.85, 1.61)
Private	16.0%		REF
Other	21.3%		1.24 (0.89, 1.73)
No Insurance	18.2%		1.03 (0.82, 1.29)
**Overall Health^5^**			
Excellent (1)	11.8%	**Chisq (3) = 462.74, p<.0001**	REF
Very Good (2)	15.8%		**1.48 (1.28, 1.70)**
Good (3)	18.4%		**1.97 (1.69, 2.29)**
Fair/Poor (4)	24.2%		**3.06 (2.62, 3.57)**
**Sexual Identity**			
Heterosexual	15.8%	**Chisq (3) = 547.3, p<.0001**	REF
Lesbian or Gay	28.0%		**1.78 (1.36, 2.33)**
Bisexual	32.2%		**1.58 (1.38, 1.81)**
Do not know/Refused/Blank	21.8%		**1.41 (1.17, 1.70)**
**Veteran (Ever served in the Armed Forces)**			
Yes (1)	12.2%	**Chisq (1) = 71.129, p<.0001**	0.93 (0.76, 1.14)
No (2)	17.6%		REF

NOTE: Level of mental illness aligns with DSM-IV criteria and is defined as having a diagnosable mental, behavioral, or emotional disorder, other than a developmental or substance use disorder. These mental illness estimates are based on a predictive model and are not direct measures of diagnostic status.

^1^ Also considered to be Any Mental Illness, but not a Serious Mental Illness

^2^ Chi-square comparing MMMI yes/no *vs.* each characteristic

^3^ Logistic regression models indicating odds of having MMMI *vs.* no MMMI

^4^ Respondents could indicate multiple insurance types; insurance types are not mutually exclusive

^5^ Respondents with unknown health data were excluded.

Source: SAMHSA, Center for Behavioral Health Statistics and Quality, National Survey on Drug Use and Health, 2021.

**Table 2:** Receipt of Mental Health Treatment by Individual Characteristics among Adults with Mild to Moderate Mental Illness, Aged 18+.

	**Past-Year Mental Health Treatment among Adults with Past-Year Mild to Moderate Mental Illness^1^** **(Weighted Percent)**	**Bivariate Chi-Square** **(df), p value**	**Adjusted Logistic Regression Model Odds Ratio** **(95% CI)**
**TOTAL**	Population N=17,484,610 Sample n=4,060 Weighted % = 17,484,610/ 42,384,329 (41.3%)	N/A	N/A
**Age Category**			
18-25	37.5%	Chisq (3) = 84.303, p=0.001	REF
26-49	41.6%		1.19 (0.97, 1.47)
50-64	48.5%		1.40 (0.99, 1.99)
65 or Older	33.5%		0.66 (0.43, 1.01)
**Sex**			
Male (1)	33.6%	Chisq (1) = 155.62, p<.0001	REF
Female (2)	46.4%		**1.60 (1.32, 1.95)**
**Race/Ethnicity**			
Not Hispanic or Latino White (1)	46.9%	Chisq (6) = 298.51, p<.0001	REF
Not Hispanic or Latino Black/African American (2)	33.1%		**0.57 (0.43, 0.75)**
Not Hispanic or Latino Native American or Alaskan Native (3)	40.8%		0.89 (0.31, 2.60)
Not Hispanic or Latino Native Hawaiian or Pacific Islander (4)	18.8%		0.27 (0.07, 1.08)
Not Hispanic or Latino Asian (5)	20.2%		**0.26 (0.19, 0.36)**
Not Hispanic or Latino More than One Race (6)	46.1%		0.95 (0.58, 1.53)
Hispanic or Latino (7)	28.2%		**0.49 (0.39, 0.63)**
**Employment**			
Full-time (1)	39.8%	Chisq (3) = 44.753, p=0.002	REF
Part–time (2)	47.0%		**1.45 (1.14, 1.85)**
Unemployed (3)	32.8%		1.04 (0.72, 1.49)
Other (4)	42.3%		**1.34 (1.06, 1.69)**
**County Type**			
Large Metro (1)	40.4%	Chisq (2) = 6.636, p=0.5631	REF
Small Metro (2)	41.4%		0.91 (0.70, 1.17)
Non-Metro (3)	44.2%		1.03 (0.74, 1.43)
**Poverty Level**			
<100% poverty threshold (1)	39.3%	Chisq (2) = 9.8321, p=0.3793	1.09 (0.77, 1.54)
100-199% poverty threshold (2)	39.3%		1.00 (0.75, 1.34)
200+% poverty threshold (3)	42.6%		REF
**Education**			
< High School (1)	28.8%	Chisq (3) = 142.83, p<.0001	**0.52 (0.35, 0.76)**
High School Graduate (2)	35.2%		**0.58 (0.46, 0.72)**
Some College (3)	44.9%		0.89 (0.70, 1.13)
College Graduate (4)	45.9%		REF
**Health Insurance^3^**			
Medicare	38.1%	Chisq (5) = 221.93, p<.0001	0.94 (0.55, 1.60)
Medicaid or CHIP	42.7%		1.00 (0.77, 1.29)
Tricare, Champus, ChampVA, VA, or Military	59.0%		1.37 (0.79, 2.38)
Private	44.1%		REF
Other	42.4%		0.94 (0.54, 1.63)
No Insurance	20.7%		**0.39 (0.25, 0.60)**
**Overall Health^2^**			
Excellent (1)	36.3%	Chisq (3) = 15.15, p=0.2164	REF
Very Good (2)	41.2%		1.19 (0.87, 1.61)
Good (3)	42.1%		**1.41 (1.07, 1.84)**
Fair/Poor (4)	42.9%		**1.62 (1.21, 2.18)**
**Sexual Identity**			
Heterosexual	17.1%	Chisq (3) = 42.83, p=0.002	REF
Lesbian or Gay	29.3%		1.18 (0.81, 1.73)
Bisexual	43.3%		**1.52 (1.20, 1.92)**
Do not know/Refused/Blank	22.5%		0.80 (0.46, 1.38)
**Veteran (Ever served in Armed Forces)**			
Yes (1)	45.8%	Chisq (1) = 4.8722, p=0.3028	1.32 (0.86, 2.00)
No (2)	41.0%		REF

NOTE: Level of mental illness aligns with DSM-IV criteria and is defined as having a diagnosable mental, behavioral, or emotional disorder, other than a developmental or substance use disorder. These mental illness estimates are based on a predictive model and are not direct measures of diagnostic status.

^1^ Also considered to be Any Mental Illness, but not a Serious Mental Illness

^2^ Respondents with unknown health data were excluded.

^3^ Respondents could indicate multiple insurance types, but were coded into a single categorical variable based on the hierarchy of type

Source: SAMHSA, Center for Behavioral Health Statistics and Quality, National Survey on Drug Use and Health, 2021.

**Table 3:** Past Year Type and Location of Mental Health Services Received by Individual Characteristics among Adults Aged 18+ with Mild to Moderate Mental Illness.

**Type and Location of Mental Health Services**	**Among Adults Aged 18+ with Past Year Mild to Moderate Mental Illness** **Weighted Percentage**	**Among Adults Aged 18+ with Past Year Mild to Moderate Mental Illness who Reported Any Past Year Treatment** **Weighted Percentage**
**ANY MENTAL HEALTH SERVICE (inpatient, outpatient, Rx, virtual)^1^**	N=17,484,610 Sample n=4,060 Weighted %: 17,484,610/ 42,384,329 (41.3%)	N=17,484,610 Sample n=4,060 Weighted %: 17,484,610/17,484,610 (100%)
**Inpatient**	1.9%	4.6%
**Outpatient**	18.6%	45.8%
Outpatient Mental Health Clinic or Center	5.1%	12.6%
Office of a Private Therapist, Psychologist, Psychiatrist, Social Worker, or Counselor - Not Part of a Clinic	11.0%	27.1%
Doctor's Office - Not Part of a Clinic	2.9%	7.2%
Outpatient Medical Clinic	1.2%	2.9%
Partial Day Hospital or Day Treatment Program	0.1%	0.3%
School or University Setting/Clinic/Center^2^	0.2%	0.4%
Some Other Place^3^	0.4%	1.1%
**Prescription Medication**	30.5%	74.1%
**Virtual Services**	25.8%	62.6%
**NONE OF THESE SERVICES**	58.7%	0%

NOTE: Mental illness aligns with DSM-IV criteria and is defined as having a diagnosable mental, behavioral, or emotional disorder, other than a developmental or substance use disorder. These mental illness estimates are based on a predictive model and are not direct measures of diagnostic status.

NOTE: Perceived unmet need for mental health services is defined as a perceived need for treatment/counseling that was not received. Perception of unmet need was asked of all respondents regardless of their mental health. Respondents with an unknown perception of unmet need information were excluded.

^1^ Respondents could indicate multiple reasons for not receiving mental health services; thus, these response categories are not mutually exclusive; mental health services, including virtual services for adults, include inpatient treatment/counseling, outpatient treatment/counseling, use of prescription medication for problems with emotions, nerves, or mental health, and virtual services. Respondents with unknown mental health services, including virtual services information, were excluded. Data were only available for those indicating a perceived unmet need for mental health services.

^2^Respondents were permitted to specify other reasons for not receiving mental health services. Reasons related to COVID-19 were collectively the most common write-in response.

^3^Respondents with unknown or invalid responses to the write-in question on Some Other Reason for Not Receiving Mental Health Services were classified as having provided a “no” response for some other reason.

Source: SAMHSA, Center for Behavioral Health Statistics and Quality, National Survey on Drug Use and Health, 2021.

**Table 4:** Reason for Not Receiving Mental Health Services in the Past Year among Adults Aged 18+ with Mild to Moderate Mental Illness among those Perceiving an Unmet Need for Care.

**Reason Did Not Receive Mental Health Services^1^**	**Aged 18+ with Past-Year Mild to Moderate Mental Illness^2^ with Past Year Perceived Unmet Need for Mental Health Care** **Weighted Percentage**
**TOTAL POPULATION**	Population N=8,434,890 Sample n=2,410 Weighted %=19.9%
Could Not Afford Cost	40.1%
Might Cause Neighbors/Community to Have Negative Opinion	10.8%
Might Have Negative Effect on Job	7.9%
Health Insurance Does Not Cover Any Mental Health Services	7.9%
Health Insurance Does Not Pay Enough for Mental Health Services	16.3%
Did Not Know Where to Go for Services	31.1%
Concerned about Confidentiality	9.8%
Concerned about Being Committed/Having to Take Medicine	9.2%
Did Not Feel the Need for Treatment at the Time	9.6%
Thought Could Handle the Problem Without Treatment	27.1%
Treatment Would Not Help	11.9%
Did Not Have Time	20.4%
Did Not Want Others to Find Out	7.2%
No Transportation/Inconvenient	3.6%
COVID-19-Related^3^	2.9%
Some Other Reason^4^	15.0%

NOTE: Mental illness aligns with DSM-IV criteria and is defined as having a diagnosable mental, behavioral, or emotional disorder, other than a developmental or substance use disorder. These mental illness estimates are based on a predictive model and are not direct measures of diagnostic status.

NOTE: Perceived unmet need for mental health services is defined as a perceived need for treatment/counseling that was not received. Perception of unmet need was asked of all respondents regardless of their mental health. Respondents with an unknown perception of unmet need information were excluded.

^1^Respondents could indicate multiple reasons for not receiving mental health services; thus, these response categories are not mutually exclusive; mental health services, including virtual services for adults, include inpatient treatment/counseling, outpatient treatment/counseling, use of prescription medication for problems with emotions, nerves, or mental health, and virtual services. Respondents with unknown mental health services, including virtual services information, were excluded. Data were only available for those indicating a perceived unmet need for mental health services.

^2^Also considered to be Any Mental Illness, but not a Serious Mental Illness.

^3^Respondents were permitted to specify other reasons for not receiving mental health services. Reasons related to COVID-19 were collectively the most common write-in response.

^4^Respondents with unknown or invalid responses to the write-in question on Some Other Reason for Not Receiving Mental Health Services were classified as having provided a “no” response for some other reason.

Source: SAMHSA, Center for Behavioral Health Statistics and Quality, National Survey on Drug Use and Health, 2021.

## Data Availability

The data supporting the findings of the article is available at https://www.samhsa.gov/data/data-we-collect/ n-sumhss-national-substance-use-and-mental-health-services-survey/datafiles/2021.

## LIST OF ABBREVIATIONS

MMMI: Mild to Moderate Mental Illness
NSDUH: National Survey on Drug Use and Health
AMI: Any Mental Illness
SMI: Serious Mental Illness
SAMHSA: Substance Abuse and Mental Health Services Administration
DMHIs: Digital Mental Health Interventions
